# Production of tropane alkaloids in *Hyoscyamus niger* (black henbane) hairy roots grown in bubble-column and spray bioreactors

**DOI:** 10.1007/s10529-013-1426-9

**Published:** 2013-12-10

**Authors:** Zbigniew Jaremicz, Maria Luczkiewicz, Adam Kokotkiewicz, Aleksandra Krolicka, Pawel Sowinski

**Affiliations:** 1 Department of Pharmacognosy, Faculty of Pharmacy, Medical University of Gdansk, Al. Gen. J. Hallera 107, 80-416 Gdańsk, Poland; 2 Laboratory of Biologically Active Compounds, Department of Biotechnology, Intercollegiate Faculty of Biotechnology, University of Gdansk and Medical University of Gdansk, ul. Kladki 24, 80-822 Gdańsk, Poland; 3 Nuclear Magnetic Resonance Laboratory, Chemical Faculty, Gdansk University of Technology, ul. Narutowicza 11/12, 80-233 Gdańsk, Poland

**Keywords:** Black henbane, Bubble bioreactors, Hairy roots, *Hyoscyamus niger*, Methyl jasmonate (elicitor), Scopolamine, Spray bioreactor, Tropane alkaloids

## Abstract

Hairy root cultures of *Hyoscyamus niger* were cultivated in shake-flasks, a bubble-column bioreactor and a hybrid bubble-column/spray bioreactor and evaluated for alkaloid production. The latter gave the highest anisodamine content (0.67 mg/g dry wt) whereas scopolamine, hyoscyamine and cuscohygrine concentrations were highest in the bubble-column reactor (5.3, 1.6 and 26.5 mg/g dry wt, respectively). Both bioreactors gave similar productivities of scopolamine (1 and 0.98 mg/l day) and cuscohygrine (5 and 5.4 mg/l day), but anisodamine productivity was 3.5-fold higher in the hybrid bioreactor (HB) (0.02 and 0.07 mg/l day, respectively). Elicitation with methyl jasmonate increased scopolamine productivity by 146 % in roots grown in the HB whereas their permeabilization with DMSO caused 4-, 5-, 25- and 28-fold increase in scopolamine, hyoscyamine, anisodamine and cuscohygrine concentrations in the growth medium. In situ extraction with Amberlite XAD-2 doubled scopolamine productivity in the hybrid reactor after 50 days.

## Introduction

Tropane alkaloids belong to widely used drugs of plant origin. Due to their anticholinergic activity, they are commonly applied as antispasmodics and mydriatics (Grynkiewicz and Gadzikowska 2008). The most valued tropane alkaloid, scopolamine, has fewer side effects and is also used in anesthetic premedication and in alleviating the symptoms of motion sickness. Given the significant demand for tropane alkaloid-rich material, researchers have sought for alternative sources of the above compounds based on in vitro cultures of Solanaceae plants, but no commercially viable systems have yet been obtained (Palazón et al. 2008).

The aim of this work was to examine the alkaloid content in hairy root cultures of *Hyoscyamus niger* (black henbane) grown under different conditions. The species was chosen because of its high potential in terms of scopolamine production, resulting from the presence of hyoscyamine 6*β*-hydroxylase (H6H) with high catalytic efficiency (Li et al. 2012), as well as the possibility of its overexpression (Palazón et al. 2008). Hairy roots were the biomass of choice in view of their fast growth, suitability for large-scale cultures (Srivastava and Srivastava 2007; Georgiev et al. 2012) and localization of tropane alkaloid biosynthesis in the roots (Palazón et al. 2008). We have examined aspects of upstream (elicitation, scale-up experiments in bioreactors) and downstream processing (permeabilization, in situ extraction). Previously, bioreactor cultures of *H. niger* hairy root were grown by Woo et al. (1996) who used submerged and mist systems. However, their studies dealt only with the production of hyoscyamine and scopolamine but not with improving the productivity of the system using elicitation or in situ extraction.

The research reported here is the first designed to increase the production of tropane alkaloids in bioreactor-grown *H. niger* hairy roots through the previously mentioned techniques. Moreover, the original *H. niger* bioreactor systems developed for the purpose of this research were evaluated, for the first time, for the production of not only hyoscyamine and scopolamine, but also anisodamine, the biosynthetic precursor of the latter, and cuscohygrine, an element of the parallel pyrrolidine alkaloid route.

## Materials and methods

### Chemicals

All reagents used for media preparation and in vitro experiments were from Sigma. Reagents used for phytochemical analyses and PCR were the same as described by Jaremicz et al. (2013) and Krolicka et al. (2010), respectively.

### Plant material

*Hyoscyamus niger* (black henbane) shoot cultures, which were subsequently used for hairy root initiation, were established from seeds (provided by the Medicinal Plant Garden of the Medical University of Gdansk) per the procedure described by Uranbey (2005), and maintained in a growth chamber [continuous light, white fluorescent tubes, (88 ± 8) μM/m^2^s; (24 ± 1) °C] on Murashige and Skoog medium (MS) supplemented with 3 % (w/v) sucrose, 0.7 % (w/v) agar and 16 μM benzyladenine (pH 5.7).

### Hairy root initiation and maintenance

**Fig. 1:**
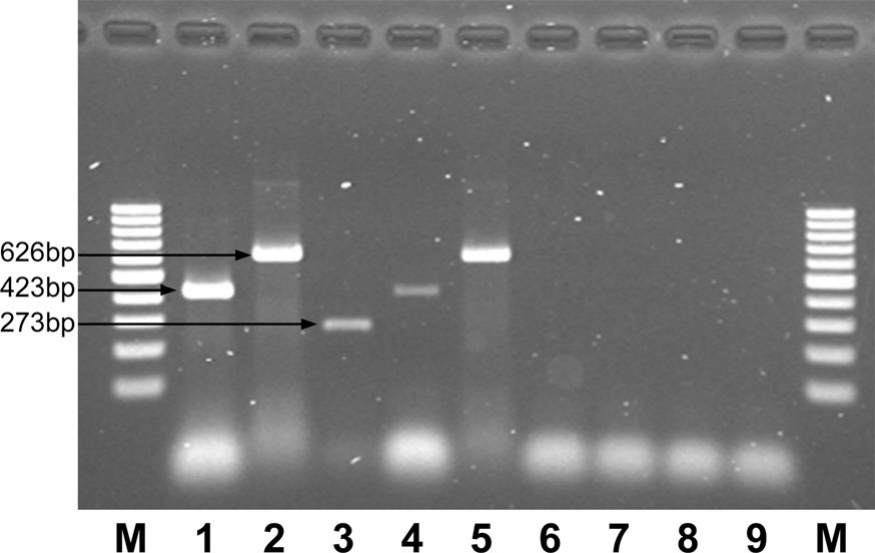
PCR analysis of *Hyoscyamus niger* roots transformed by *Agrobacterium rhizogenes* LBA1334. M. Marker GeneRuler 100 bp DNA ladder; PCR reactions were performed using as a target DNA isolated from: *1* *A. rhizogenes* LBA1334 + primer *rolB* as a positive control, *2* *A. rhizogenes* LBA1334 + primer *rolC* as a positive control, *3* *A. rhizogenes* LBA1334 + primer *virG* as a positive control, *4* Hairy roots of *H. niger* + primer *rolB*, *5* Hairy roots of *H. niger* + primer *rolC*, **6** Hairy roots of *H. niger* + primer *virG*, *7* Non - transformed roots of *H. niger* + primer *rolB* as a negative control, *8* Non - transformed roots of *H. niger* + primer *rolC* as a negative control, *9* Non-transformed roots of *H. niger* + primer *virG* as a negative control. *Arrows* show amplified fragments of *rolB* (423 bp; *lanes* *1*, *4*, *7*), *rolC* (626 bp; *lanes* *2*, *5*, *8*) and *virG* (273 bp; *lanes* *3*, *6*, *9*) genes

For hairy root induction, leaf explants from *H. niger* shoot cultures were infected with *Agrobacterium rhizogenes* LBA1334 (derivative of C58 with pRi1855 plasmid, also harbouring the binary plasmid pBIN-m-gfp5-ER) (Baranski et al. 2006), incubated in the dark onto solidified (0.7 % w/v agar), phytohormone-free MS medium supplemented with 3 % (w/v) sucrose and after 24 h transferred on the same medium supplemented with antibiotics (cefotaxim and amoxicillin, 500 mg/l each). After 3 weeks, the hairy roots were excised, transferred to liquid medium of the same composition and subcultured in 4-week intervals. After six passages, sterile hairy roots were transferred to antibiotic-free medium and the transformation was confirmed by PCR in accordance with Krolicka et al. (2010) (Fig. 1).

The obtained cultures were maintained in the dark, at 24 ± 1 °C, in 125 ml Ehrlenmeyer flasks (shaken at 120 rpm, 25.4 mm stroke) containing 25 ml phytohormone-free MS medium (pH 6.0) supplemented with 3 % (w/v) sucrose. Hairy roots were subcultured every 30 days. For shake flask (SF) and bioreactor experiments, the 1.6:100 and 2.5:100 (w/v) inoculum:medium ratio was applied. Hairy roots were comminuted to <20 mm fragments prior to bioreactor inoculation.

### Elicitation, permeabilization and in situ extraction experiments

For elicitation, the respective amount of stock solution of methyl jasmonate (MeJ) at 2.5 and 25 mM in 96 % (v/v) ethanol was added (1 ml) to the growth medium by sterile filtration to give 0.1 or 1 mM.

For permeabilization, DMSO was sterile-filtered into the hairy root culture to give 2, 4 or 20 % (v/v).

Amberlite XAD-2 (20–60 mesh) was pretreated by stirring 50 ml resin with chloroform (100 ml, 15 min) followed by methanol (100 ml, 15 min) and redistilled water (3 × 100 ml, 3 × 15 min). XAD-2 was subsequently packed into a cartridge (see “Bioreactor experiments” section) and autoclaved together in the bioreactor.

### Bioreactor experiments

**Fig. 2:**
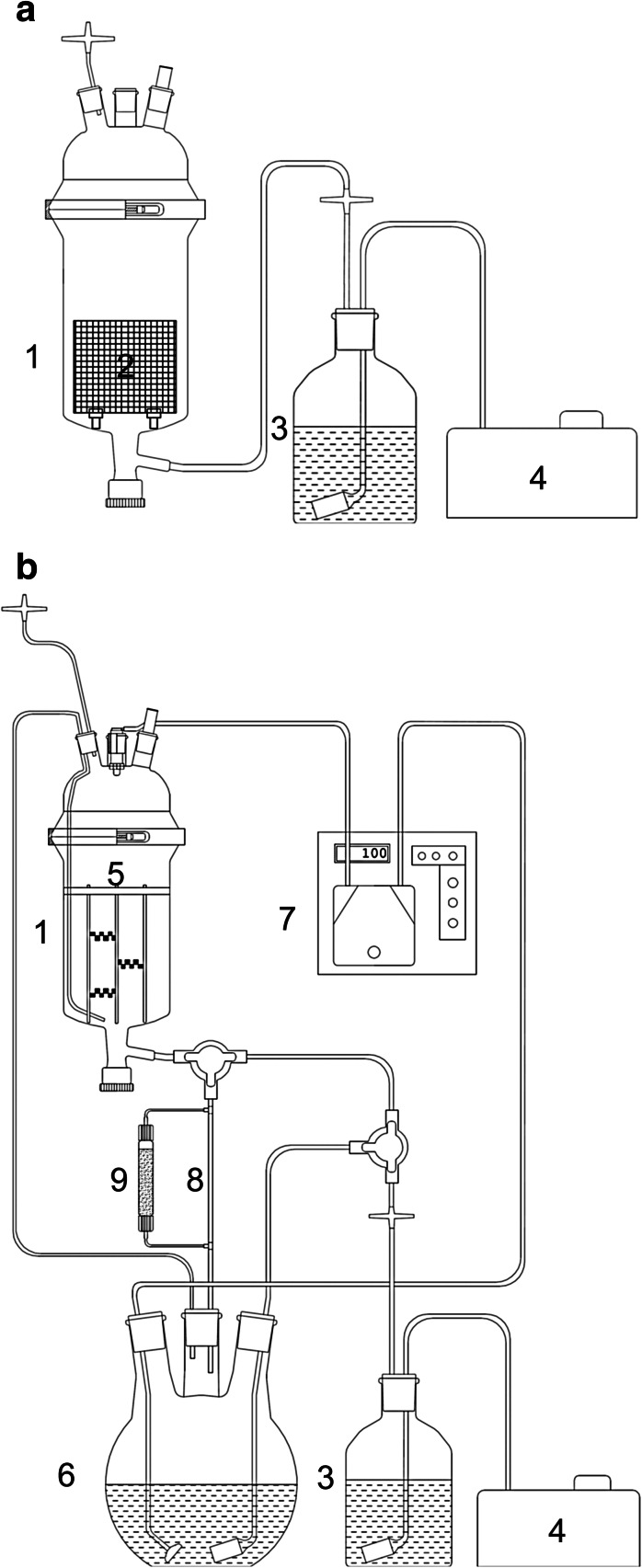
Schematic diagrams of: **a** BCB and **b** HB. *1* growth vessel, *2* immobilization basket, *3* air humidifier, *4* air pump, *5* immobilization system (details presented in Fig. 2), *6* medium reservoir, *7* peristaltic pump, *8* medium drain, *9* cartridge with XAD-2 resin (in situ extraction experiment only)

The experiments included two bioreactors: bubble column (BCB) and bubble-column/spray hybrid bioreactor (HB), which were steam sterilized (120 °C, 0.1 Mpa) before use. The BCB (Fig. 2a) consisted of a cylindrical glass vessel (120 ID × 200 mm) equipped with 3 mm ID air inlet and 95 × 80 mm stainless steel basket (8 mm mesh) for root immobilization. The initial working volume was 600 ml and the subsequent 200 ml portions of the growth medium were added to the bioreactor on day 20 and day 40 of the experiment. Hairy roots were comminuted to <20 mm fragments prior to inoculation. Aeration rate was kept at a constant 0.8 vvm.

**Fig. 3:**
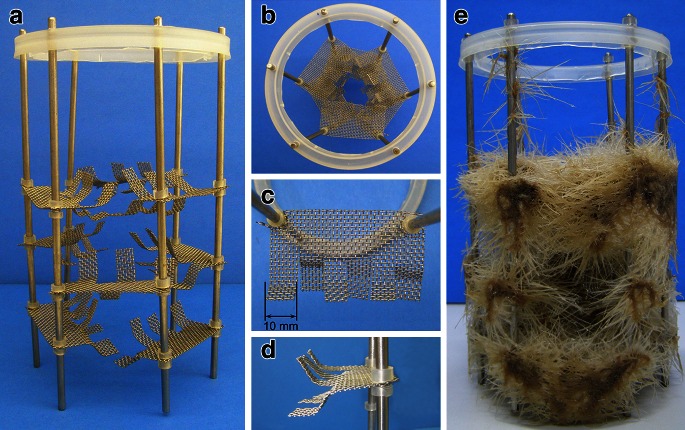
Details of root immobilization system used in HB: **a** system overview, **b** top view, **c** single mesh strap top view, **d** side view, **e** system with roots after 30 days of culture

The HB (Fig. 2b) consisted of a cylindrical glass vessel of the same dimensions as the BCB. It was equipped with a drain valve, a medium reservoir and a multi-tier stainless steel scaffolding composed of rods and 1 mm mesh straps (3 × 6 mm), notched and bent as presented in Fig. 3a–d. For biomass immobilization, the growth vessel was filled with medium (1,500 ml working volume), inoculated with comminuted (<20 mm) hairy roots, and the culture was maintained for 7 days in BCB mode. The medium was subsequently drained to the reservoir tank and the bioreactor was switched to spraying mode. The medium was dispersed at 100 ml/min using the 1.5 size, TN-type hydraulic nozzle (Spraying Systems Co, Wheaton, US-IL) in 200 ml portions, at 10 min intervals. Growth medium in the reservoir was aerated at a constant 0.8 vvm and the air then passed upwards the growth vessel before leaving the bioreactor. For in situ extraction studies, the HB was modified by placing a cartridge (20 mm ID × 200 mm) filled with XAD-2 (50 ml) in the drainage (Fig. 2b), so that the growth medium was filtered through the resin bed during each spraying cycle.

The biomass and medium samples from both bioreactor types were collected after unloading the installation on the respective day of experiment.

### Sample preparation and analysis of alkaloids

Samples for phytochemical analyses were prepared in accordance with a standard procedure for the isolation of alkaloids based on different solubilities of their free base and salt forms in aqueous and organic solutions. Alkaloid content in biomass and media samples was determined by HPTLC with densitometric detection. The details of the procedure were described by Jaremicz et al. (2013).

### Statistical analysis

The statistical analysis of the experimental data included the analysis of variance (ANOVA) and *t* test, at probability level of 0.05. The analyses were performed using SigmaStat 3.5 software (Systat Software, Chicago, USA).

## Results and discussion

### Effect of cultivation mode on biomass growth and alkaloid production

**Fig. 4:**
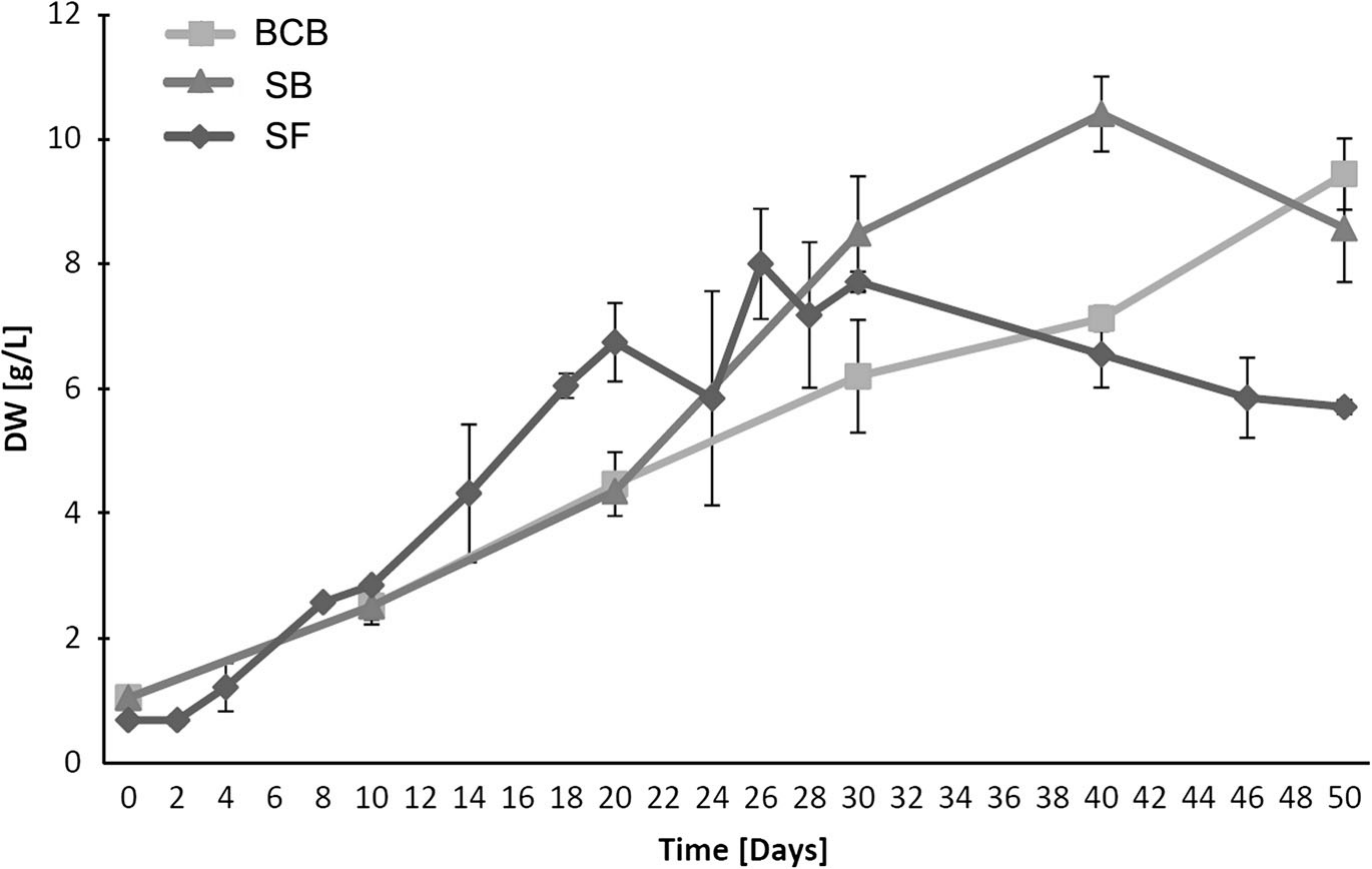
Changes in dry wt of *H. niger* hairy root cultures grown in BCB, HB and SF. Values represent the mean of 3 for SF and 2 for BCB, HB samples ± SD

**Fig. 5:**
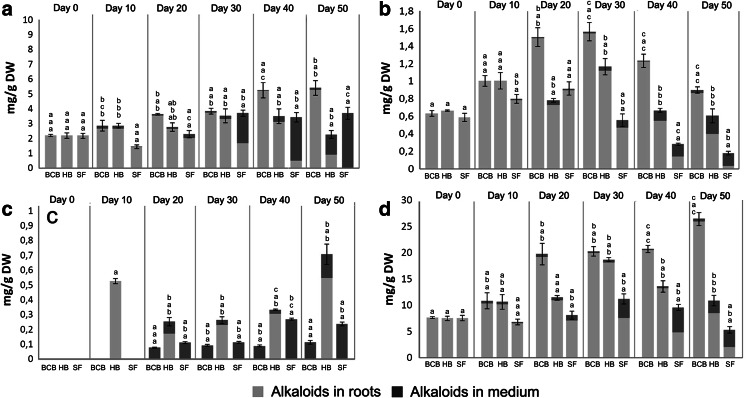
Changes in: **a** scopolamine, **b** hyoscyamine, **c** anisodamine, **d** cuscohygrine concentrations in *H. niger* hairy root cultures grown in BCB, HB, and SF. Values represent the mean of 3 samples for SF and 2 samples for BCB and HB, error bars refer to total culture (roots + growth medium) content of the respective alkaloids. *Bars* assigned with *different letters*, corresponding to alkaloid content in total culture, growth medium and roots (from *top* to *bottom*, respectively), are statistically different based on Tukey test (*p* < 0.05)

The growth profiles of *H. niger* hairy roots cultivated in different conditions are presented in Fig. 4. Maximum biomass (8 g/l) in shake-flasks (SF) was achieved on day 26 and decreased thereafter, likely as a result of nutrient depletion and/or impaired O_2_ supply (Kim et al. 2002). Alkaloids in SF-grown hairy roots was comprised of cuscohygrine (major component), followed by scopolamine, hyoscyamine, and anisodamine (Fig. 5). The production of the first three metabolites corresponded to biomass growth, with maximum concentrations reached on 20th (scopolamine and hyoscyamine) or 30th day (cuscohygrine). From 30th day onwards, increased release of the above compounds into the growth medium was observed, which corresponded with the decline growth phase (Figs 4, 5). This was also true in the case of anisodamine which, however, was present only in small amounts (<0.3 mg/g dry wt) and was stored exclusively extracellularly throughout the growth period.

*Hyoscyamus niger* hairy roots were subsequently cultivated in bioreactors, to evaluate the effects of scale-up and altered mixing/aeration conditions on biomass growth and alkaloid content. The bubble column bioreactor (BCB) was selected for the experiments because of its simple and reliable construction, as well as its applicability for hairy root cultivation (Wilhelmson et al. 2006; Min et al. 2007). The growth vessel was equipped with a stainless steel basket for root immobilization (Fig. 2), a modification previously shown to improve growth rate of roots by shortening the lag phase (Cusido et al. 1999). To prevent the culture from entering the decline phase and to provide more medium volume for the roots, the BCB was run in fed-batch mode.

As presented in Fig. 4, stationary and decline phases were eliminated, resulting in linear growth throughout the experiment. Different kinetics of biomass growth in BCB were reflected by altered profiles of alkaloid accumulation. Maximum concentrations of scopolamine, hyoscyamine and cuscohygrine were achieved later than in SF and were respectively 50, 60 and 140 % higher (Fig. 5). Considering that there is a positive correlation between O_2_ supply/aeration rate and alkaloid biosynthesis in root cultures of Solanaceous plants (Williams and Doran 2000; Wilhelmson et al. 2006; Min et al. 2007), a possible explanation of this phenomenon is the improved aeration in BCB. Also, differently than in gyratory cultures, only small amounts (1–4 %) of these compounds were found in the growth medium confirming that alkaloid release in SF most likely resulted from root necrosis. Interestingly, altered cultivation conditions did not influence the accumulation profile of anisodamine which was present only in the growth medium just as in the case of SF cultures (Fig. 5).

Furthermore, the decision to cultivate *H. niger* hairy roots in the gas-phase bioreactor, was taken as such systems are considered to provide better O_2_ and nutrients supply than conventional air-sparged vessels (Srivastava and Srivastava 2007; Georgiev et al. 2012). To provide optimal utilization of reactor volume and more efficient liquid drainage in comparison to horizontal supports (Williams and Doran 2000), the growth vessel was equipped with a vertical root immobilization system (Fig. 3). The bioreactor was run in hybrid mode (Kim et al. 2002; Srivastava and Srivastava 2007) in order to uniformly distribute root biomass. Thus, bubble-column operation (root immobilization phase) was followed by spray application of growth medium.

As expected, HB- and BCB-grown roots showed comparable growth rates during the first phase of the experiment, when both systems were run in bubble-column mode (Fig. 4). However, since day 20 HB provided faster growth, with maximum biomass yield achieved on day 40, i.e. 10 days earlier than in BCB. Despite the observed increase in biomass growth rate, maximum concentrations of the respective alkaloids (with the exception of anisodamine) in HB-grown roots were lower than in BCB (Fig. 5).

The experiment confirms that transferring the roots to gas-phase bioreactor is often insufficient to increase the production of tropane alkaloids. According to Williams and Doran (2000), liquid hold-up in the root bed was the probable cause of the lowered alkaloid content, as it was shown to impede oxygen transfer to the biomass. Since the cultivation of roots in HB stimulated root growth but not alkaloid content, it can be concluded that higher growth rate resulted not from improved O_2_ supply, but from more dispersed and uniform distribution of inocular biomass, which is considered a key factor determining the performance of gas-phase bioreactors (Williams and Doran 2000).

Despite obvious differences in the patterns of biomass growth and alkaloids production (Figs. 4, 5), HB and BCB yielded similar productivities of cuscohygrine and scopolamine (Table 1). Tropane alkaloid productivity of both bioreactor systems was lower in comparison to SF, which was due to higher root growth rates in gyratory cultures during the first half of the experiment (Fig. 4). However, this was not true in the case of anisodamine which was produced in the highest amounts in HB. Both bioreactors also provided increased production of cuscohygrine, resulting in higher overall alkaloid productivity (Table 1). In comparison to experiments by Woo et al. (1996) which employed stirred-tank and mist bioreactors, the achieved biomass yields were respectively 2 and threefold higher for submerged (BCB vs. stirred-tank) and gas-phase systems (HB vs. mist bioreactor). In our work we obtained productivities of scopolamine and hyoscyamine respectively 2 and threefold higher than those reported by Woo et al. (1996).

**Table 1:** Alkaloid productivity in *H. niger* hairy roots grown in: SF, BCB and bubble-column/spray HB

Experimental conditions	Day of the culture	Productivity (mg/l day)					
		Scopolamine	Hyoscyamine	Anisodamine	Cuscohygrine	Tropane alkaloids	Total alkaloids
SF	30^f^	1.28 ± 0.07	0.21 ± 0.03	0.04 ± 0.002	4 ± 0.36	1.53 ± 0.08	5.53 ± 0.94
BCB	50^f^	1 ± 0.09	0.17 ± 0.007	0.02 ± 0.002	4.99 ± 0.25	1.19 ± 0.07	6.18 ± 0.94
HB	30^f^	0.98 ± 0.13	0.31 ± 0.03	0.07 ± 0.006	5.36 ± 0.12	1.36 ± 0.11	6.72 ± 1.14
HB^a^	26	0.62 ± 0.06	0.15 ± 0.005	0.05 ± 0.006	2.65 ± 0.1	0.82 ± 0.55	3.47 ± 0.49
HB^b^	50	0.38 ± 0.04	0.1 ± 0.01	0.12 ± 0.01	1.89 ± 0.18	0.60 ± 0.05	2.49 ± 0.57
HB + MeJ^c^	26	1.58 ± 0.006	0.37 ± 0.02	0.06 ± 0.003	6.19 ± 0.4	2.01 ± 0.01	8.21 ± 0.38
HB + DMSO^d^	30	0.47 ± 0.09	0.47 ± 0.02	0.18 ± 0.04	2.03 ± 0.91	1.12 ± 0.07	3.42 ± 0.98
HB + XAD-2^e^	50	0.76 ± 0.001	0.05 ± 0.004	0.1 ± 0.008	2.42 ± 0.19	0.91 ± 0.01	3.33 ± 0.2

Values represent the mean of 3 for SF and 2 for BCB, HB samples ± SD

^a^Control for elicitation experiments in HB

^b^Control for in situ extraction experiments in HB

^c^Hairy roots elicited with 1 mM methyl jasmonate on the 26th day

^d^Hairy roots permeabilized with 20 % (v/v) dimethyl sulfoxide on the 30th day

^e^Culture subjected to in situ extraction with Amberlite XAD-2 resin from 30th to 50th day

^f^The day on which maximum productivity of the respective system was obtained

### Elicitation, permeabilization and in situ extraction experiments

Features which determine the suitability of an in vitro system for commercial production of natural compounds are productivity and location of secondary metabolites. These features are affected by different experimental strategies, with elicitation being one of the most common (Georgiev et al. 2012). In the present work, hairy roots of *H. niger* were treated with MeJ, which stimulated the biosynthesis of tropane derivatives in root cultures of black henbane (Zhang et al. 2007) and other plants from the nightshade family (Kim et al. 2002). MeJ also acts as a permeabilizing agent triggering the efflux of alkaloids into the growth medium (Palazón et al. 2008).

**Fig. 6:**
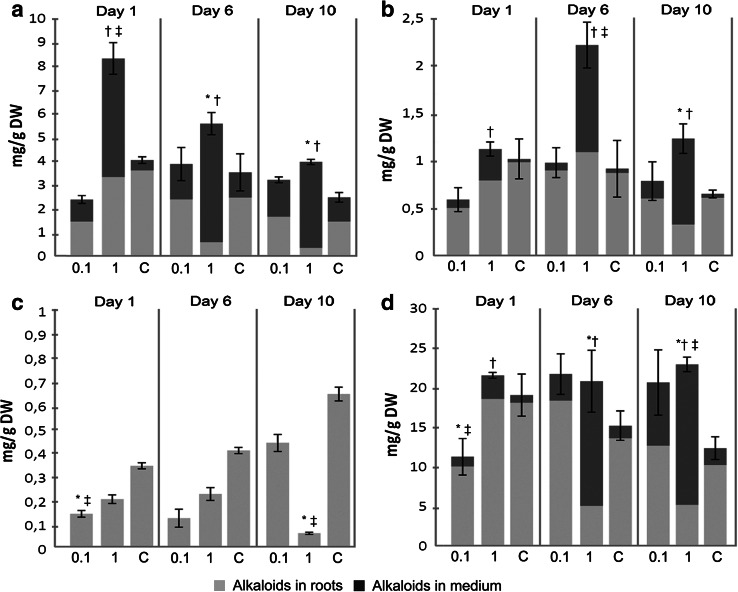
Effects of different concentrations of methyl jasmonate (mM), added on day 26, on the concentrations of **a** scopolamine, **b** hyoscyamine, **c** anisodamine and **d** cuscohygrine in SF-grown *H. niger* hairy root cultures. Values represent the mean of 3 samples, *error bars* refer to total culture content of the respective alkaloids. *Symbols* indicate significant difference (Holm-Sidak test, *p* < 0.05) in alkaloid content in roots (*asterisk*), growth medium (*dagger*) and total culture (*double dagger*) in comparison to control group (*C* cultures supplemented with ethanol only)

**Fig. 7:**
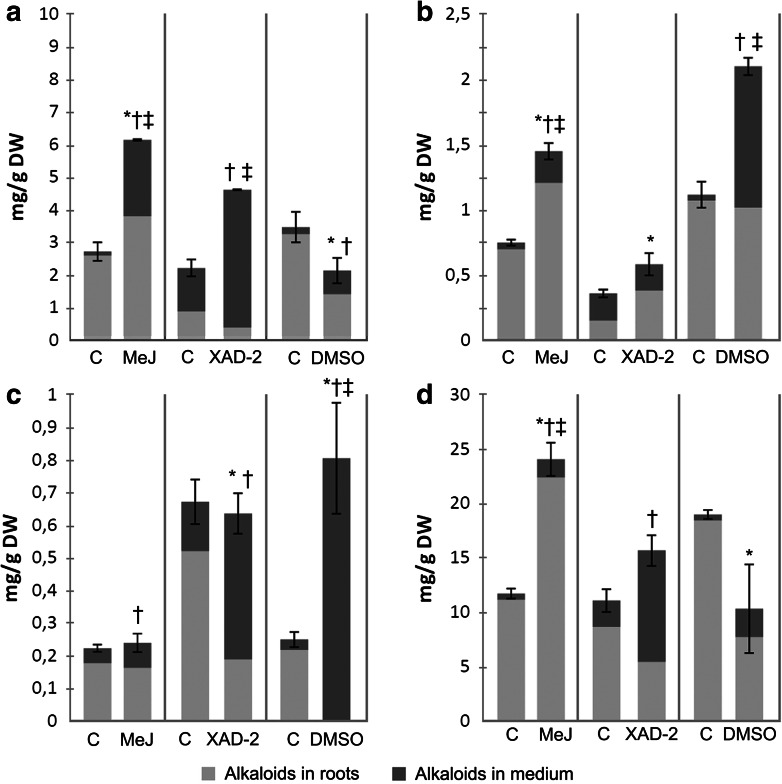
Effects of different concentrations (% v/v) of dimethyl sulfoxide, added on day 30, on the concentrations of **a** scopolamine, **b** hyoscyamine, **c** anisodamine and **d** cuscohygrine in SF-grown *H. niger* hairy root cultures. Values represent the mean of 3 samples, *error bars* refer to total culture content of the respective alkaloids. *Symbols* indicate significant difference (Holm-Sidak or Dunn’s test, *p* < 0.05) in alkaloid content in roots (*asterisk*), growth medium (*dagger*) and total culture (*double dagger*) in comparison to control group (*C* culture grown without the presence of DMSO)

As shown in Fig. 6, the addition of MeJ at 1 mM caused a significant increase of scopolamine concentration in the growth medium as well as over 100 % increase in total scopolamine content after 24 h. Given that scopolamine is the most valuable constituent of the examined alkaloid set, the above approach was applied to bioreactor-grown roots. The experiment was run in the HB, where scopolamine productivity was comparable to other systems and also gave the highest total alkaloid yield (Table 1). Moreover, our work confirmed previous studies claiming that sprinkle bioreactor provides more favorable conditions for hairy root elicitation in comparison to SF (Kuźma et al. 2009) since the treatment of HB-grown roots with MeJ caused not only over 100 % increase in scopolamine content, similarly to the experiment in SF, but also ca. 100 % increase in the concentrations of hyoscyamine and cuscohygrine (Figs. 6, 7). As seen in Table 1, the changes in alkaloid content corresponded to increased system productivity in terms of both tropane and total alkaloids.

**Fig. 8:**
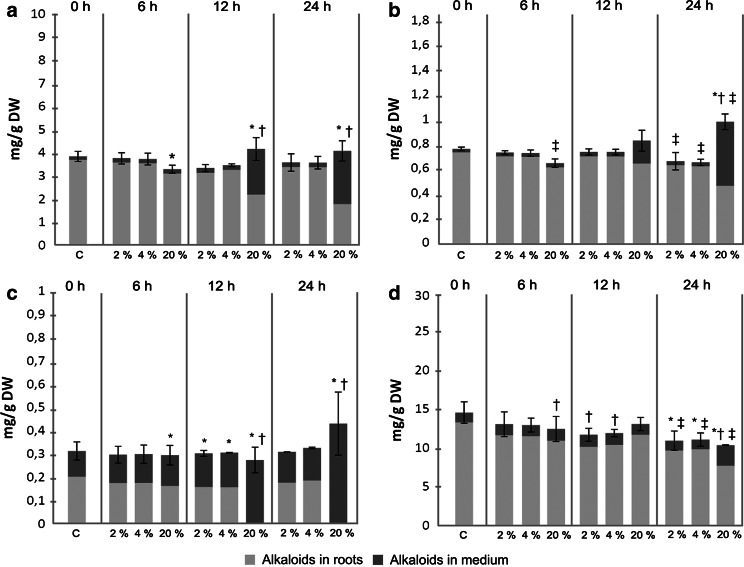
Effects of different experimental strategies on the concentrations of **a** scopolamine, **b** hyoscyamine, **c** anisodamine and **d** cuscohygrine in HB-grown *H. niger* hairy root cultures. Values represent the mean of 2 samples, *error bars* refer to total culture content of the respective alkaloids. *Symbols* indicate significant difference (*t* test, *p* < 0.05) in alkaloid content in roots (*asterisk*), growth medium (*dagger*) and total culture (*double dagger*) in comparison to control. *MeJ* hairy roots elicited with 1.0 mM methyl jasmonate on the 26th day, collected after 24 h; *DMSO* hairy roots permeabilized with 20 % (v/v) dimethyl sulfoxide on the 30th day, collected after 24 h; *XAD-2* culture subjected to in situ extraction with Amberlite XAD-2 resin from 30 to 50 days, collected on 50th day; *C* control group—cultures grown for the same period of time without the presence of MeJ, DMSO or XAD-2

Since extracellular alkaloids are generally considered easier to recover (which can be done either by liquid–liquid partitioning (Jaremicz et al. 2013) or in situ extraction using polymeric resins (Cusido et al. 1999), hairy roots of *H. niger* were treated with DMSO, which proved to be an effective permeabilizing agent in previous studies on plant cell cultures (Luczkiewicz and Kokotkiewicz 2012). No permeabilization experiments on black henbane hairy roots have been conducted so far. Figure 8 depicts the effects of various concentrations of DMSO on the accumulation of alkaloids in SF-grown roots. Substantial increase in tropane alkaloid content in the medium was achieved only after 24 h incubation of the roots with 20 % (v/v) DMSO. An analogous experiment conducted in HB provided mixed results. The concentrations of hyoscyamine and anisodamine in the growth medium were increased, and so were their productivities. On the other hand, the permeabilization of HB-grown roots failed to trigger the efflux of scopolamine into the growth medium (Fig. 7).

The in situ extraction experiments were aimed at both enabling easier product recovery and improving system productivity. According to previous studies, the removal of tropane alkaloids from the medium by absorption on XAD-2 resin can stimulate their production by preventing feedback inhibition (Cusido et al. 1999). Unlike the elicitation and permeabilization experiments, the in situ extraction procedure was not applied to SF cultures due to the expected difficulties in separating roots from the adsorbent and the mechanical stress generated by resin beads. On the other hand, HB with an immobilized root bed and XAD-2-filled cartridge (Fig. 2b) proved to be suitable for the planned experiment. The idea was to improve scopolamine productivity during the last 20 days of the growth period, which were characterized by high biomass yield (Fig. 4) and increasing concentrations of extracellular scopolamine (Fig. 5). The growth medium was allowed to drain freely through the Amberlite bed from day 30 to day 50 of the experiment. The result was a 100 % increase in total scopolamine concentration and productivity on day 50 (Fig. 7; Table 1).

**In summary**, *H. niger* hairy root cultures can be maintained in both submerged (bubble column) and gas-phase bioreactors and give comparable tropane alkaloid productivities, which were higher than previously reported (Woo et al. 1996). Elicitation and in situ extraction with Amberlite XAD-2 resin of roots grown in the hybrid bubble-column/spray bioreactor were the most successful in stimulating alkaloid biosynthesis. This bioreactor system, as well as the productivity-enhancing procedures, were applied for the first time to black henbane in vitro cultures. Moreover, this is the first report on *H. niger* hairy root cultures dealing with the accumulation of anisodamine, a scopolamine precursor, and cuscohygrine, a product of parallel biosynthetic pathway. We can now assume that a combination of elicitation and in situ extraction can be applied in further studies on hairy roots of *H. niger*. Considering that both of the examined bioreactors (i.e. submerged and gas-phase systems) are potentially scalable up to 10,000 l (Georgiev et al. 2012), the established cultures can be further evaluated as an in vitro source of tropane alkaloids.
